# How We Adopted the Uniform System of Accounts

**Published:** 1911-08-12

**Authors:** 


### HOW WE ADOPTED THE UNIFORM SYSTEM OF ACCOUNTS.

BY THE SECRETARY TO THE EAST SUFFOLK AND IPSWICH HOSPITAL.

The Uniform System of Hospital Account Keeping and its adoption at my hospital is described below with some diffidence, considering that my experience in an official capacity with voluntary hospitals has extended over a short period. I will preface my remarks by stating that the East Suffolk and Ipswich Hospital, of 134 beds, has recently been improved, enlarged, and modernised at a cost of over £20,000. The district served by the hospital comprises the borough, town, and port of Ipswich, and the major portion of the administrative county of East Suffolk, consisting of 245 country parishes and including three small seaside resorts.

### First Acquaintance with Uniform System.

My first acquaintance with the Uniform System came about by my discovery that the accounts of this hospital were not scheduled in the Income and Expenditure Tables of "Burdett's Annual for 1910," and an inquiry at the publishers elicited the information that the accounts were not presented in a form that could be used for comparison with other hospitals. A careful scrutiny of the reports of the large hospitals throughout the Kingdom revealed the fact that as to those which did not present their accounts on the Uniform System no two were precisely alike, and consequently useful comparisons were practically impossible. Before recommending my Board to adopt the Uniform System, I went carefully into the matter and discussed the subject with several officials of provincial hospitals, some of whom advocated its entire adoption, others its adoption with certain reservations, and a few its rejection.

### A Preliminary Objection.

One of the objections raised was the great increase in bookkeeping consequent upon altering a system from the simple method of "Receipts and Payments" to "Income and Expenditure," entailing the inclusion of liabilities in the year's expenditure. Another objection was the new method of counting patients—that is to say, counting the actual individuals, not the number of tickets. But perhaps the greatest objection to the system was on the question of work entailed in recording the in- and out-patient statistics. Dealing with these objections *seriatim*, I would say that I have not found the Income and Expenditure Account more difficult to keep than that of a Receipts and Payments Account, and it must be clear to the minds of all thinking persons that a Receipts and Payments Account cannot reveal a true statement of the year's transactions. In this connection I would also say that the Board holds a meeting at the close of our financial year to pay all current accounts, hence the liabilities included in the balance-sheet are necessarily small. The method of enumerating patients certainly meant working on an entirely different basis, if it did not duplicate the work, as the "letter or ticket" system was and is in force here, as in most provincial hospitals, and the figures previously given were of the tickets rather than of the patients. As to the last objection, that of the work entailed in recording statistics, this was a factor which had to be met.

However, I considered the benefits likely to accrue would easily outweigh the objections, and I had no hesitation in recommending my Board to adopt the Uniform System, and the Board unanimously resolved to do so, subject to the financial year closing on May 31, as hitherto, instead of on December 31, as there were valid reasons why the change of date should not take place immediately.

### My Own View.

Be it far from me to criticise the authors of the Uniform System, but my first impression was that the Revenue or Income and Expenditure Account appeared to differ from the usual form in which such accounts are presented, in that the order of debit and credit appears to be transposed. Another point was that, although the liabilities are included in the Expenditure Account, the value of stores in hand is not included in the balance-sheet. No doubt the authors were of opinion that its inclusion would necessitate more work than the average secretary would be likely to undertake.

With regard to the statistical tables, the space allocated under Out-patients 2 (*a*) for the number of patients on books at the beginning of year seems somewhat unnecessary in a hospital like ours, as it is almost impossible to deduce correctly these figures, and I notice that this space has been left blank in the reports of some of our largest hospitals, although their system and manner of recording out-patients leave nothing to be desired. It also struck me as singular that in the In-patients Statistical Form there was no heading such as "Total number of In-patients treated to a conclusion," although this appears to form the basis of calculating the cost of two important items later on. But in the absence of any rule against increasing the number of statistics given, I may be pardoned for the inclusion of this item, as well as some other special notes in my report. I have also found it a convenience to give a statement of income and expenditure on capital account for the year as distinct from the prescribed forms.

### The Real Difficulties.

The first difficulty to be met was the all-important question of the number of patients, and to meet this I instituted a daily register of the number of in-patients showing the number of beds occupied at a certain hour each day, with forms to be filled up daily by the sister in charge of each ward. The out-patient department proved a more difficult matter, as a system of counting new cases and attendances had to be instituted for the first time. I found it necessary to get the porters to collect the major part of the data for this daily return, and to those who are about to adopt the Uniform System I would say that in my opinion this is an important item and one which encroaches on the time of someone, whoever that person may be.

The greatest practical difficulty hitherto met, and one which is likely to continue, is the dissection of the drug accounts as between in- and out-patients. As we have one dispensary only, which serves both the hospital and the out-patient department, the difficulty of our position is clear.

With regard to the accountancy side of the question, I did not find it necessary to discard any of the existing account books, save the general ledger and petty-cash book, nor was it necessary to purchase any additional books. One of my friends (a hospital official) explained to me that the very foundation of the Uniform System was an elaborate analysis journal, but personally I do not believe in analysis journals and do not keep one, as I prefer to keep my accounts as do a great many Local Government bodies, and my ledger, which is specially ruled for the purpose, gives all the information required for this system for the use of my Board and to the satisfaction of the auditor, the Income and Expenditure Account, balance-sheets, and statistical tables shown in the annual report being extracted direct from the general ledger.

### What the Changes Meant.

I have already stated that the "letter or ticket" system is in vogue here, and the practice in obtaining a record of the numbers has been the same as in many other provincial hospitals—that is, to let a ticket figure as an individual and to assess the cost of an out-patient empirically at a given figure. This makes the result of the first complete year's statistics under the new system a little puzzling to the subscribers and not a little disappointing to the officials. The number of both in- and out-patients has increased greatly during the past two years, but the results shown are as follows:—

| | Uniform System. Years ending May 31. 1911. | Old System. Years ending May 31. 1909. |
|---|---|---|
| Number of in-patients admitted during the year | 1,265 | 1.565 |
| Total number of new out-patients... | 9,571 | 12,768 |
| Average number of days each in-patient was resident | 31.8 | 22.77 |
| Average total cost of each in-patient | £5 9 3 | £3 9 8¼ |
| Average total cost of each in-patient per week | £1 4 0½ | £1 1 5 |

A recount of the in-patients admitted in 1909 reveals the fact that the number, if taken on the Uniform basis, is 1,160 instead of 1,565.

In the Income and Expenditure Account the payments were allocated under five heads, and it therefore became necessary to reconstruct the previous four or five years' expenditure so as to form a basis of local comparison.

I am not quite convinced in my own mind that in every instance where the Uniform System has been adopted the number of patients has been given strictly in accordance with the rules laid down, but I certainly think that they should be.

The investments had been entered at their original cost, and, as the bulk of them had been held for many years, considerable depreciation had taken place. Under the old system there was no balance-sheet, and consequently the investments were not brought into account, so I embraced the opportunity of writing these down to their market value on bringing them into the new balance-sheet. To those about to adopt the Uniform System, whose investments have not been written down in recent years, I would suggest this course, as to my mind it is better to show the capital account lower rather than higher than its market value, which will keep the hospital on the right side in case a heavy sale of stock is necessary to meet enlargements or other extraordinary expenditure.

### An Irrefragable Rule.

If it be essential that one of the rules of the Uniform System should be carried out to the letter, that rule must be irrefragable which enacts that the lists of annual subscriptions, etc., should be cast and agreed with the income and expenditure account. This, I find, has not been done by a number of hospitals which purport to submit their accounts on the Uniform System. The answer to this will probably be that some subscribers have not paid their subscriptions at the time of closing the accounts, and that it is impossible to agree the totals until it is ascertained whether they will pay or not, while the exclusion of their names from the list might prejudice the hospital. To this I would say that, as to the majority of the subscribers, the amounts should have been paid at the beginning of the year, and as to those who pay at the half-yearly stage, if they do not pay within six months and after repeated applications, no amount of badgering will induce them to pay when they are further in arrear and no longer entitled to letters of recommendation. For this reason I have omitted such from my list.

### A Point for Provincial Secretaries.

In justice to the provincial hospitals I submit that the clerical staff and the number of porters generally allowed to them is so small in comparison with the number of those of the London hospitals that statistics cannot be recorded so well, nor even such elaborate statements be produced as are current in the larger hospitals of the Metropolis.

### What are the Advantages?

From an officer's point of view the advantages of the Uniform System of Accounts are manifold. The hospital expenditure is light or heavy by comparison only, and hitherto the only reliable basis of comparison is that formulated in the Uniform System. In dealing with the Boards of Management of the voluntary hospitals of our country one meets the keenest business and professional men of our time, and the secretary who would maintain his position must be competent to discuss the financial relations of his hospital to those of other hospitals similarly situated. Therefore I recommend the Uniform System to those secretaries who have not yet adopted it, and, having adopted it, advise them to carry it out to the letter, as far as circumstances may permit.

The Subscribers' View.

After all, it is the subscribing public who are the interested parties in our annual reports, and the many expressions of appreciation I have received from those who have made a careful study of the new system, as opposed to the old, leaves no doubt in my mind that uniformity of accounts and statistics is the very keynote of our financial dealings with the philanthropic public.

One of our perennial problems, particularly in the Provinces, is to make the annual report readable, and I wonder sometimes whether the usefulness of the Uniform System of Accounts for this purpose has been emphasised enough. In at least one way our own report has benefited: it has become, in my opinion, a more systematically compiled book of reference; it is therefore more readable and handy to the inquirer, and, in short, a genuinely better book. It emphasises the old statistical system which separates the donations from the annual subscriptions, that makes not only the separate items in the grand total analysed more readily, but also allows the subscribers to find their own names more quickly and with a more exact relation to their particular gifts than hitherto. It may seem a small thing to the layman gently to flatter the donor's or annual subscriber's susceptibilities. Hospital officers know better, and as sometimes a hospital supporter has given to his institution in more than one capacity, his name may appear in more than one place, and, if it is entitled to do so, all experience teaches that he is encouraged by finding that recognised in the report.

Assistance.

I cannot conclude without expressing my indebtedness to the officials of the Incorporated Association of Hospital Officers for continual assistance in dealing not only with questions arising out of the Uniform System of Accounts, but those which affect an officer in any department of a hospital. In my opinion, this Association is essentially one for the interchange of social and official experience of hospital officers, and therefore it does not clash with any other society or association, having a sphere it has made its own.

